# Non-alcoholic fatty liver disease and pregnancy complications among Sri Lankan women: A cross sectional analytical study

**DOI:** 10.1371/journal.pone.0215326

**Published:** 2019-04-12

**Authors:** Rasika Pradeep Herath, Shirom R. Siriwardana, Chanil D. Ekanayake, Vikum Abeysekara, Sajith U. A. Kodithuwakku, Himali P. Herath

**Affiliations:** 1 Department of Obstetrics and Gynaecology, Faculty of Medicine, University of Kelaniya, Ragama, Sri Lanka; 2 Department of Anatomy, Faculty of Medicine, University of Kelaniya, Ragama, Sri Lanka; 3 Department of Clinical Sciences, General Sir John Kotelawala Defence University, Ratmalana, Sri Lanka; 4 Department of Community Medicine, Faculty of Medicine, University of Colombo, Colombo, Sri Lanka; East Tennessee State University, UNITED STATES

## Abstract

**Background:**

Non-alcoholic fatty liver disease (NAFLD) is the commonest cause of liver disease worldwide and is the hepatic manifestation of metabolic syndrome. Effects of NAFLD on pregnancy is still unclear with few studies showing an association to gestational diabetes and pre-eclampsia. We aimed to describe the association between the NAFLD and pregnancy complications. This is the first study, to our knowledge, in a South Asian population.

**Method:**

A cross sectional analytical study was done in Teaching Hospital, Ragama, Sri Lanka. Women carrying a singleton pregnancy, admitted for delivery were assessed for NAFLD with liver ultrasound scan. Data were extracted from interviewer administered questionnaire and antenatal and inpatient records. Pregnancy complications and labour outcomes were compared between the women with NAFLD and women without NAFLD (non-NAFLD).

**Results:**

Out of the 573 women who participated, 18.2% (n = 104) were found to have NAFLD. Out of them, 58 (55.8%), 32(30.8%), and 14(13.5%) had fatty liver grade 1,2 and 3 respectively. Women with NAFLD were 2 times more likely to develop gestational hypertension and pre-eclampsia compared to the women in the non-NAFLD group, after adjusting for BMI, age and Hyperglycaemia in pregnancy [Adjusted OR 2.09, (95% CI 1.07–4.10)]. There was no association between the grade of steatosis and a composite outcome of gestational hypertension and pre-eclampsia, within the NAFLD group. Composite outcome of gestational diabetes mellitus and diabetes in pregnancy diagnosed during pregnancy was a significant complication in the NAFLD group compared to non-NAFLD group in the bivariate analysis (27.2% vs 17.7%; p<0.05), but the significance disappeared after adjusting for confounders. The current study did not demonstrate a significant association between NAFLD with preterm labour, caesarean section rate, low birth weight, and Apgar score of the baby.

**Conclusion:**

Women with NAFLD had a 2-fold higher risk of developing gestational hypertension and pre-eclampsia during pregnancy compared to women without NAFLD, after controlling for other confounding variables.

## Introduction

Non-alcoholic fatty liver disease (NAFLD) is the commonest cause of liver disease worldwide and is the hepatic manifestation of metabolic syndrome, frequently co-existing with obesity, dyslipidaemia and insulin resistance [1,2]. It represents a range of diseases from non-alcoholic fatty liver, non-alcoholic steatohepatitis, and ultimately liver cirrhosis [3]. NAFLD is defined as the presence of hepatic steatosis, either by imaging or histology, in the absence of significant alcohol consumption and other secondary causes of hepatic fat accumulation [4].

The prevalence and grade of NAFLD varies widely depending on the population screened and the screening tool used for the diagnosis. The prevalence of histologically-defined NAFLD was 20% and 51% in two different studies involving potential living liver donors [4,5]. The reported prevalence of NAFLD when defined by liver ultrasound ranged between 17% and 46% depending on the population studied {South America (31%), middle East (32%), USA (23%), Europe (24%)}[6,7,8]. In Sri Lanka, a community based study demonstrated an incidence of 37.5% of NAFLD among women in 2008[9]. Furthermore, a recent study in 2017 revealed an incidence of 8.7% of NAFLD among adolescents in an urban population of Sri Lanka [10].

Interestingly, the interaction between NAFLD, obesity and insulin resistance appears to vary depending on an individual’s race and ethnicity [2]. Asian individuals with NAFLD have a lower BMI than those in western countries and significant proportion have low insulin resistance [11]. This evidence suggests that Asian populations have different genetic and environmental susceptibility to NAFLD, thus emphasizing the need to study its implications in pregnancy.

There is only scarce evidence in the literature about the prevalence of NAFLD among pregnant women and pregnancy outcome. The first study of NAFLD in pregnancy was published in 2007 as a cause of abnormal liver function tests during pregnancy [12]. There is evidence to show that the diagnosis of NAFLD during the first trimester is associated with hyperglycaemia in mid pregnancy in Canadian, Korean and Egyptian women, though they used different diagnostic criteria [13,14,15]. A retrospective analysis of birth registry in Sweden found 110 women (delivered between 1992 and 2011) with NAFLD diagnosed prior to pregnancy, and reported to have increased risk of gestational diabetes (GDM), caesarean section, extreme preterm birth, preeclampsia (PE) and low birth weight [16]. However in the latter study, the women who were categorized as non-NAFLD did not undergo any form of imaging or histology to exclude the disease. We could not find any studies describing the relationship of NAFLD and pregnancy complications in South Asia.

This study aimed to identify the prevalence of NAFLD among pregnant women and pregnancy outcomes in the University Obstetrics and Gynaecology unit, at North Colombo Teaching Hospital, Ragama, Sri Lanka.

## Materials and methods

### Design, setting, participants and method

A cross sectional analytical study was done from 18^th^ August to 28 October 2017 in University Obstetrics and Gynecology unit, North Colombo Teaching Hospital, Ragama, Sri Lanka. This hospital is one of the main tertiary care units in the western province of Sri Lanka, close to its capital, Colombo. The hospital has three Obstetrics and Gynaecology units. University Obstetrics and Gynecology Unit, where the study was done, had 3316 deliveries with a caesarean section rate of 34.8% in 2017, and manned by five specialist obstetricians.

Consenting women carrying a singleton pregnancy admitted for delivery (or caesarean section) were considered to the study. Data were collected with an interviewer administered questionnaire, with special regard to past medical history and alcohol consumption. Antenatal records were observed and data regarding women’s health status at booking, weight, blood pressure, and results of blood sugar screening were gathered. The participants height, weight and blood pressure were checked and inpatient notes (bed head tickets) were observed for pregnancy complications. All participants had blood sent for Alanine Aminotransferase (ALT), Aspartate Aminotransferase (AST) and underwent an abdominal ultrasound scan (USS) during admission. Once the baby was delivered, additional data about delivery were collected from the inpatient notes (bed head tickets).

Women with known liver disease (Viral and autoimmune hepatitis, cholangitis and inborn errors of metabolism), alcohol consumption and exposure to medications causing hepatic steatosis (corticosteroids, amiodarone, tamoxifen and valproate) were excluded. None of the participants had a history of acute fatty liver of pregnancy. All women who participated confirmed that they never had hepatitis nor consumed alcohol or smoked.

Ultrasonography was done by a specialist radiologist and a senior registrar in obstetrics and gynaecology, who was trained to perform liver USS. One operator performed scans during the morning session and saved the images, while the second operator independently assessed the images in the evening. When there was a discrepancy, they discussed and agreed upon the diagnosis. CHISON iVis 60 EXPERT unit was used to acquire gray scale images using a low frequency 2–5 MHz multi frequency convex transducer. Multiple transverse and longitudinal images were taken showing the hepato-diaphragmatic interface, hepato-renal interface, inferior vena cava with hepatic vein confluence, portal vein and its branches, porta hepatis and gall bladder. The field of view was adjusted to include the diaphragm on the longitudinal images. Focal zones, gain settings and field of view was adjusted to obtain the maximum clarity [17]. The operators were not aware of the results of liver function tests of the patients.

### Outcome measures

Parity was defined as number of previous births after 24 weeks. If the current pregnancy is the first time a pregnancy has continued beyond 24 weeks she was considered as primigravida, and if she has one previous pregnancy that continued beyond 24 weeks she was considered as para 1.

Monthly family income was recorded in Sri Lankan rupees. We did not have the pre-pregnancy bodyweight, therefore we used the bodyweight at booking visit to calculate the weight gain in pregnancy and booking BMI. The participants BMI was grouped as underweight (<18.5 kg/m^2^), normal (18.5–24.99 kg/m^2^), or overweight (≥25 kg/m^2^), according to the recommendations of the Maternal Care Package of Sri Lanka [18]. It is known that being overweight and being obese increases the risk of developing gestational hypertension (GH), PE and GDM, and thus both groups were analyzed as one identified group with a BMI ≥25 kg/m^2^ [19,20].

Ultrasonography is used for diagnosis of NAFLD. The presence of NAFLD was identified by the detection of bright echogenic patterns within the liver (we have excluded the alcohol consumption and secondary causes for steatosis during recruitment) [4].

During USS liver echo pattern was categorized into four grades [21,22].

A. Grade -0 (Normal)

Liver parenchyma has a homogeneous echo texture with fine low level echos, Liver echogenicity equal to or slightly greater than that of the normal renal cortex and spleen.

B. Grade -1(Mild steatosis)

Liver echogenicity is slightly increased and clear delineation of hepatic and portal vein walls.

C. Grade II (Moderate steatosis)

Liver echogenicity is moderately increased, obscuring the echogenic walls of hepatic and portal vein branches. Echogenic line of diaphragm is well visualized

D. Grade III (Severe steatosis)

Marked increase in hepatic echogenicity, poor visualization echogenic walls of hepatic vessels, poor visualization diaphragm or obscure the clear delineation of diaphragm and liver, poor visualization of posterior portion of the right lobe.

Every woman whose liver echo pattern falling into Grade I, II and III were diagnosed to have NAFLD (cases). Women who were in Grade 0 were identified as Non-NAFLD or controls.

Antenatal care package in Sri Lanka recommends performing blood sugar screening tests at booking visit and at 24–28 weeks of pregnancy. Most of the women undergo 75g oral glucose tolerance test, while minority undergo only a fasting blood sugar test depending on the available resources. Cumulative results of these screening tests of the participants were analyzed. We identified women into three groups; Diabetes diagnosed before pregnancy (preexisting diabetes), Gestational diabetes mellitus (GDM) and Diabetes in pregnancy (DIP), diagnosed for the first time during pregnancy according to current guidelines [20]. GDM and DIP during pregnancy were diagnosed according to FIGO 2015 recommendations for the high risk population of Indian subcontinent [20]. Either plasma glucose of 5.1−6.9 mmol/L (92−125 mg/dL) or fasting (or non-fasting) 2-hour plasma glucose value following 75-g oral glucose between 7.8 and 11.0 mmol/L (140 and 199 mg/dL) was considered as GDM [20]. DIP was diagnosed with fasting plasma glucose ≥7.0 mmol/L (126 mg/dL); and/or 2-hour plasma glucose ≥11.1 mmol/L (200 mg/dL) following a 75-g oral glucose load; or random plasma glucose ≥11.1 mmol/L (200 mg/dL) in the presence of symptoms of diabetes [20]. Women who were known to have diabetes before the pregnancy (either on medications and or on diet) were considered as Diabetes diagnosed before pregnancy (pre-existing diabetes). We used the term ‘Hyperglycaemia diagnosed during pregnancy’ in this article to denote the composite of GDM and DIP diagnosed during pregnancy.

Chronic hypertension is diagnosed when hypertension was present at the booking visit or before 20 weeks or if the woman was already on antihypertensives when referred to maternity services [23]. Gestational hypertension (GH) was defined as new hypertension (diastolic blood pressure 90–99 mmHg, systolic blood pressure 140–149 mmHg) presenting after 20 weeks without significant proteinuria [23]. The diagnosis of Pre-eclampsia (PE) was considered as patients with hypertension presenting after 20 weeks with significant proteinuria [23]. Delivery of a baby before 37^+0^ weeks was considered as preterm delivery [24]. Birth weight less than 2500g was identified as low birth weight [25].

### Data analysis

Baseline characteristics of women in the NAFLD and non-NAFLD groups were described using descriptive statistics. Variables were tested for normality using the Kolmogorov Smirnov test. Frequencies and percentages were used to summarize categorical variables and means (SD) and medians (IQR) were used to summarize continuous variables. Group means/medians were compared between NAFLD and non-NAFLD groups using Student t test and Mann-Whitney U test respectively. Categorical variables between NAFLD and non-NAFLD were compared using Chi square test.

Binary logistic regression analysis was used to explore the associations between NAFLD and development of hyperglycaemia diagnosed during pregnancy and composite outcomes of GH and PE, controlling for other predictors. The mean values of ALT and AST were compared between NAFLD grades with general linear model adjusting for composite of GH and PE.

### Ethical considerations

The study was approved by the Ethical Review Committee, Faculty of Medicine, University of Kelaniya (Reference: P/153/06/2017)). Written informed consent was taken from participants. Women with NAFLD were educated about the importance of regular exercises and dietary control.

## Results

There were 744 women delivered during the period and 573 eligible women were recruited. Out of the study subjects 104 (18.2%), were diagnosed to have NAFLD. Out of the 104 women with NAFLD, 58 (55.8%) had grade 1 fatty liver disease while 32 (30.8%) had grade II fatty liver. Only 14 (13.5%) were diagnosed having grade III fatty liver. Basic characteristics of the two groups are shown in the Table 1.

**Table 1:** Characteristics of the NAFLD and non-NAFLD group.

Variable		NAFLD N = 104	Non-NAFLD N = 469	p value
Maternal age at delivery (years)	Mean (SD)	31.13(4.95)	29.28(5.60)	0.002
Ethnicity	Sinhala	N = 95 (91.3%)	N = 447(95.3%)	0.11
	Other	N = 9(8.7%)	N = 22(4.7%)	
Parity	Primigravida	N = 38(36.5%)	N = 224(47.8%)	0.115
	Para 1	N = 44(42.3%)	N = 164(35.0%)	
	Para 2 or more	N = 22(21.2%)	N = 81(18.0%)	
POA at booking visit (weeks)^a^	Median (IQR)	7.54 (6.1–8.6)	7.19 (6.0–8.9)	0.44
Maternal education^b^	Primary	N = 4(3.9%)	N = 4 (0.9%)	0.008
	Secondary	N = 89 (87.3%)	N = 444(94.9%)	
	Tertiary	N = 9(8.8%)	N = 20(4.3%)	
Monthly family income in Sri Lankan Rupees^c^ (USD = 149.4 rupees in September 2017)	≤ 30000	N = 25(27.5%)	N = 109(28.1%)	0.564
	30001–39999	N = 10(11.0%)	N = 46(11.9%)	
	40000–49999	N = 20(22.0%)	N = 71(18.3%)	
	50000–64999	N = 14(15.4%)	N = 86(22.2%)	
	≥ 65000	N = 22(24.2%)	N = 76(19.6%)	
Pre-existing diabetes	Yes	N = 4(3.8%)	N = 8(1.7%)	0.157*
	No	100(96.2%)	461(98.3%)	
Chronic Hypertension	Yes	3(2.9%)	4(0.9%)	0.116*
	No	101(97.1%)	465(99.1%)	
BMI (kg/m^2^)^d^	Mean (SD)	26.41 (5.05)	23.34(4.60)	<0.001
BMI Category at booking visit ^d^	<18.5	4(3.9%)	66(14.2%)	<0.001
	18.5–24.99	34(33.3%)	255(55.0%)	
	>25	64 (62.7%)	143 (30.8%)	
Number of T1 miscarriages	0 miscarriages	80(76.9%)	362(77.2%)	0.76
	1–2 miscarriages	22(21.2%)	102(21.7%)	
	≥3 miscarriages	2(1.9%)	5(1.1%)	
History of T2 miscarriages	Yes	2(1.9%)	8(1.7%)	1*
	No	102(98.1%)	461(98.3%)	
Booking SBP^e^	Mean (SD)	112.33(11.34)	110.98(10.36)	0.286
Booking DBP^e^	Mean (SD)	72.79(7.13)	71.27(7.4)	0.084
AST^f^	Mean (SD)	22.36(8.53)	21.03(8.06)	0.136
ALT^g^	Mean (SD)	13.77(6.78)	12.41(6.67)	0.066

ALT, Alanine aminotransferase; AST, Aspartate aminotransferase; BMI, Body mass index; DBP, Diastolic blood pressure in mmHg; IQR, Interquartile range; NAFLD, Non-alcoholic fatty liver disease; POA, Period of amenorrhoea; SBP, Systolic blood pressure in mmHg; T1, First trimester; T2, Second trimester

*Fisher’s exact test

^a^ Data available for 82 cases and 378 controls

^b^ Data available for 102 NAFLD cases and 468 controls

^c^ Data available for 91 NAFLD cases and 388 controls

^d^ Data available for 104 NAFLD cases and 464 controls

^e^ Data available for 86 NAFLD cases and 374 controls

^f^ Data available for 101 NAFLD cases and 464 controls, blood tested during the admission for delivery

^g^ Data available for 101 NAFLD cases and 463 controls, blood tested during the admission for delivery

Women in the NAFLD group were significantly older than the non-NAFLD group. The mean booking BMI of NAFLD women was 26.41 kg/m^2^ (SD 5.05) while in the non-NAFLD group it was 23.34 kg/m^2^ (SD 4.6) (p < .001). When the BMI categories were compared, 64 (62.7%) of NAFLD women were found to be overweight or obese, while 143 (30.8%) in the non-NAFLD group were overweight or obese (p < .001).

There was no significant difference between the two groups in terms of pre-existing diabetes and chronic hypertension. Past history of first and second trimester miscarriages were not significantly different between the NAFLD and non-NAFLD groups (Table 1).

Complications and outcomes of the current pregnancy are shown in Table 2. The mean weight gain during pregnancy in the NAFLD group was 8.20 Kg (SD 4.59), while the non-NAFLD group had a mean weight gain of 9.21 Kg (SD 4.66) (p <0.05). Among women without preexisting diabetes, there were 5 (5.4%) women diagnosed with DIP diagnosed during pregnancy and 20(21.7%) women with GDM in the NAFLD group while there were 10 (2.3%) and 65 (15.2%) women in the non-NAFLD group respectively. Hyperglycaemia diagnosed during pregnancy (composite outcome of GDM and DIP diagnosed during pregnancy) accounted for 25 (27.2%) in the NAFLD group and 75 (17.5%) among non-NAFLD group (p<0.05). But, this significant association between Hyperglycaemia during pregnancy and NAFLD group disappeared in the bivariate analysis after adjusting for confounders (Table 3).

**Table 2:** Pregnancy complications and outcomes in the NAFLD and non-NAFLD groups.

Variable	Parameter	NAFLD (N = 104)	Non-NAFLD (N = 469)	p value
Weight gain in pregnancy^a^	Mean (SD)	8.20 (4.59)	9.21 (4.66)	<0.05
Glycaemic status at 28 weeks^b^	DIP diagnosed during pregnancy	N = 5(5.4%)	N = 10(2.3%)	0.068
	GDM	N = 20(21.7%)	N = 65(15.2%)	
	Normal	N = 67(72.8%)	N = 353(82.5%)	
Hyperglycaemia diagnosed during pregnancy^b^	Composite of GDM and DIP diagnosed during pregnancy	N = 25(27.2%)	N = 75(17.5%)	<0.05
	Normal	N = 67(72.8%)	N = 353 (82.5%)	
Composite of GH and PE	Yes	N = 18(17.3%)	N = 37(7.9%)	<0.01
	No	N = 86(82.7%)	N = 432(92.1%)	
PE	Yes	N = 2 (1.9%)	N = 4(0.9%)	0.299*
	No	N = 102(98.1%)	N = 465(99.1%)	
POA at delivery	Mean (SD)	N = 37.77(4.32)	N = 38.54 (2.20)	<0.05
POA at delivery	<37 weeks	N = 17(16.3%)	N = 58(12.4%)	0.276
	≥37 weeks	N = 87(83.7%)	N = 411(86.9%)	
Mode of delivery	Vaginal delivery	N = 66(64.1%)	N = 296(63.1%)	0.862
	Instrumental delivery	N = 3(2.9%)	19(4.1%)	
	Caesarean Section	N = 34(33.0%)	N = 154(32.8%)	
Apgar score at 5 minutes after birth	0–6	N = 2(2.0%)	N = 10(2.1%)	1.0*
	>7–10	N = 99(98.0%)	N = 459(97.9%)	
Birth weight of the baby (g)^c^	Mean (SD)	2949.3(582.6)	2897.9(577.2)	0.87
	<2500	N = 19 (18.4%)	N = 99 (21.1%)	0.77
	2500–3499	N = 69 (67.0%)	N = 310 (66.1%)	
	>3500	N = 15(14.6%)	N = 60(12.8%)	

DIP, Diabetes in pregnancy; GDM, Gestational diabetes, GH, Gestational Hypertension; NAFLD, Non-alcoholic fatty liver disease; POA, Period of amenorrhoea; PE, pre-eclampsia

*Fisher’s exact test

^a^ data available for 103 NAFLD cases and 464 controls

^b^12 women with pre-existing diabetes were not considered. Missing data 41 [8 (7%) for NAFLD cases 33 (7.7%) for controls]. There were 15 women with DIP diagnosed during pregnancy and 85 women with GDM

^c^Data available for 103 NAFLD cases and 469 controls

**Table 3:** Crude and adjusted odds ratios for development of composite outcome of GH and PE, and composite outcome of developing DIP and GDM during pregnancy.

	NAFLD	Non-NAFLD	Crude odds ratio (95% CI)	Adjusted odds ratio (95% CI)
Composite outcome of GDM and DIP diagnosed during pregnancy^a^				
Yes	N = 25	N = 75	1.756(1.04–2.96)	1.304(0.75–2.27)
No	N = 67	N = 353	1	
Composite outcome of GH and PE^b^				
Yes	N = 18	N = 37	2.444(1.33–4.49)	2.093(1.07–4.1)
No	N = 86	N = 432	1	

DIP, Diabetes in pregnancy; GDM, Gestational diabetes; GH, Gestational hypertension; NAFLD, Non-alcoholic fatty liver disease; PE, Pre-eclampsia

^a^ Women with pre-existing diabetes were not considered. Missing data 41 [8 (7%) for NAFLD cases 33 (7.7%) for controls]. The model was adjusted for mothers age >35, BMI≥25, and presence of GH and PE

^b^ The model was adjusted for mothers age >35, BMI≥25, and presence of DIP and GDM diagnosed during pregnancy.

There was a higher proportion of women with composite of Gestational Hypertension and PE (18, 17.3% Vs 37, 7.9%, p<0.01) in the NAFLD group. We could not see an association between the level of steatosis and a composite outcome of gestational hypertension and PE, within the NAFLD group. There was a significant association between composite of GH and PE in women with NAFLD (Adjusted odds ratio 2.09, 95% CI 1.07–4.1) independent of BMI>25, age > 35 years and having Hyperglycaemia diagnosed during pregnancy (Table 3).

The women in the NAFLD group have delivered slightly earlier [37.77 weeks (SD 4.32) Vs 38.54 (SD 2.2)] than the controls (p<0.05). 17(16.3%) of women in the NAFLD group had preterm deliveries while 58(12.4%) delivered preterm among the controls (p = 0.276). There was also no significant difference between caesarean, vaginal delivery and instrumental delivery rates among the two groups (p = 0.862). The mean birth weight of babies of NAFLD women was 2.95 kg (SD 0.58) while it was 2.90 kg (SD 0.58) in the non-NAFLD women(p = 0.87). The two groups did not show a significant difference with regard to proportion of small for gestation babies, normal weight babies and large babies (p = 0.77). There was also no difference among babies in the two groups regarding Apgar at 5 minutes (p = 1).

The mean ALT level in the NAFLD group was 13.77 U/L,(SD 6.78) and was slighter higher than the non-NAFLD group (12.41U/LSD 6.67), and the difference observed was not significant (Table 1). However, when the NAFLD group was stratified based on the degree of steatosis (ultrasound grading) and compared with mean ALT level, the ALT level increased as the ultrasound grading of liver steatosis increased, after adjusting for composite of GH and PE (p<0.05)(Adjusted R^2^ = 0.023)(Table 4). Serum AST levels did not show a significant difference among the groups (Table 4).

**Table 4:** Relationship between ALT and AST levels and the grade of fatty liver.

	Grade of fatty liver	N	Mean	SD	F value	p Value
AST value^a^	0 (non-NAFLD)	464	21.030	8.056	1.653	0.176
	I	57	21.837	8.609		
	II	31	21.871	8.441		
	III	13	25.846	8.214		
ALT value^b^	0 (non-NAFLD)	463	12.415	6.666	2.727	0.043
	I	57	12.602	4.608		
	II	31	14.710	9.697		
	III	13	16.638	5.637		

ALT, Alanine aminotransferase; AST, Aspartate aminotransferase

^a^ Data available for 565 subjects

^b^ Data available for 564 subjects

## Discussion

To our knowledge, this is the first study on NAFLD during pregnancy in South Asia, where there is a rising pandemic of metabolic syndrome. Further, to our belief, it is the first study to describe pregnancy outcomes of women with NAFLD identified during the third trimester close to delivery. In our study we found a prevalence of NAFLD of 18.2% among the pregnant population. This is lower than the previously reported figure of 37.4% among non-pregnant women in Sri Lanka [9]. But the mean age of this non-pregnant population was 52.5 years and had a higher mean BMI (27.1 kg/m^2^) [9]. We could find only two studies describing the prevalence of NAFLD among pregnant women which showed a prevalence of 17.6% in a Canadian population and 18.4% in a Korean population [13,14]. The finding of higher BMI in the NAFLD group in our study is probably explained by its association of the condition to increasing obesity [9].

The mean period of amenorrhoea (POA) at delivery in the NAFLD group was significantly lower than the non-NAFLD group. This could be due to higher proportion of women with DIP, GDM, gestational hypertension and PE among the NAFLD group, where early delivery is indicated. We found no association between preterm birth and NAFLD. The evidence in literature is divided as one study found an association between extreme preterm birth (<32 weeks) and the other did not find an association [15,16].

There are only two studies we could find in the literature analyzing the association of hypertensive disorders in pregnancy with NAFLD. Hagstrom et al in study in Sweden, found an increased risk of PE in women with NAFLD, but the significance disappeared when adjusted for BMI. But their study, done using Swedish Medical Health Register and National Patient Register, identified only110 cases with NAFLD out the 1,960,416 (0.0006%) of the women studied [16]. It is very likely that there were lot of women with NAFLD included in the non-NAFLD group, who never had USS for screening [16]. This would have had a major impact in the final outcome. Mousa et al found an increased association with PE and NAFLD, though not adjusted for the risk of obesity or BMI. The generally quoted incidence of preeclampsia is 3–7% in nulliparous and 1–3% in multiparous women [26]. But in the study of Mousa et al, there were 14% of women with PE in the non-NAFLD group making their finding less generalizable to other populations [15]. Our study found that women with NAFLD have increased risk of developing composite outcome of gestational hypertension and PE during pregnancy, independent of BMI >25, age>35 and Hyperglycaemia diagnosed during pregnancy (while PE alone was not significantly associated). NICE guidelines identifies following risk factors to consider for commencing Aspirin by 12 weeks of pregnancy, in order to reduce the risk of developing PE: hypertensive disease during a previous pregnancy, chronic kidney disease, autoimmune disease such as systemic lupus erythematosus or antiphospholipid syndrome, type 1 or type 2 diabetes, chronic hypertension, being a primipara, age of 40 years or older, pregnancy interval of more than 10 years, BMI of 35 kg/m^2^ more at first visit, family history of pre-eclampsia and multiple pregnancy [23]. As adjusted odds ratio of NAFLD for GH and PE is 2.09, we recommend considering NAFLD to be included to the above list of risk factors after further research.

Only a few studies have tried to discern the relationship between the GDM and NAFLD. The above mentioned Swedish study of Hagstrom et al, reported increased risk of developing GDM in women NAFLD diagnosed prior to pregnancy, but the association was not clear among the obese women. But the criteria used for the diagnosis in their study was not mentioned [16]. De Souza et al in their prospective study done in Canada in a multi-ethnic population, found an ultrasound diagnosis of NAFLD at 11–14 weeks of pregnancy to be associated with an increased risk of developing GDM according to IADPSG (International Association of Diabetes and Pregnancy Study Group) criteria [13, 27]. Mousa et al also found an increased risk of GDM among women with NAFLD but had not adjusted the risk against confounders; age and BMI [15]. They used American Diabetes Association (ADA) criteria as fasting plasma glucose ≥7 mmol/L (126 mg/dL) and/or 2-h plasma glucose ≥11.1 mmol/L (200 mg/dL) after a 75 g oral glucose load for the diagnosis of GDM. Lee et al in their study done in Korea demonstrated risk of developing GDM (diagnosis of GDM required two or more elevated glucose levels, i.e. ≥5.3 mmol/l for fasting glucose, ≥10 mmol/l for 1 h glucose, ≥8.6 mmol/l for 2 h glucose and ≥7.8 mmol/l for 3 h glucose, following a glucose load of 100g) was significantly increased in women diagnosed to have NAFLD at 10–14 weeks of pregnancy and was positively correlated with the severity of steatosis [14,28]. Further, their study confirmed the above association even when NAFLD is defined by non-invasive steatosis indices such as Fatty liver index and Hepatic steatosis index[14]. Our study showed an association between NAFLD and Hyperglycaemia diagnosed in pregnancy, but the significance of the association disappeared when adjusted for BMI and age. This is in consistent with findings of the Swedish study of Hagstrom et al. South Asians are at increased risk of GDM and DIP, and our cutoff values for diagnosis of GDM and DIP were lower than most of the other studies [20]. The diversity of the criteria used to diagnose diabetes would have contributed to differences noted above. Further we assume that at lower blood glucose thresholds, relationship between BMI, insulin resistance, age, and ethnicity may be stronger contributors to Hyperglycaemia than NAFLD. Interestingly, the relationship between NAFLD, obesity and insulin resistance has been shown to vary depending on an individual’s race/ethnicity also, though the exact mechanisms are not well understood [2,29].

Insulin resistance (IR) has a major role in the development of hepatic steatosis [29]. In addition, steatosis itself can promote insulin resistance commencing a self-propagating vicious cycle. The most accepted model is the initiation of IR peripherally in the adipose tissues [30,31]. High BMI and obesity indirectly depict the expanding adiposity in the body. The excessive accumulation of fat in adipocytes promotes an increase in oxidative stress and allow a low-grade inflammatory state in adipose tissues. This results in release of proinflammatory cytokines such as tumour necrosis factor-α and interferon-γ from macrophages and lymphocytes, further promoting IR [32]. Further, the spillover of free fatty acids leads to accumulation of fat in ectopic sites such as liver and muscle. At these sites ectopic fat induces further IR, propagating the cascade of obesity, NAFLD and IR [33]. This mechanism explains the strong association of IR (thus resulting GDM and DIP) and increased BMI among patients with hepatic steatosis. Closer biochemical interrelated relationship between obesity and IR may have masked the relationship of NAFLD to dysglycaemia in pregnancy in our study. Further research is recommended to shed more light on the association of NAFLD and IR in pregnancy.

NAFLD and metabolic syndrome is known to be associated with hypertension in nonpregnant women. Many mechanisms have been proposed to link insulin resistance or hyperinsulinaemia to hypertension, including stimulation of the sympathetic nervous system, sodium absorption by hyperinsulinaemia, and impaired insulin stimulation of vasodilation [34,35]. How NAFLD contributes to GH and PE is yet to be understood. In addition to our study showing a significant association between NAFLD and composite of GH and PE, independent of high BMI, the association of rising BMI with GH and PE is well established [36]. PE is a low-grade inflammatory state with the balance between vasoconstrictive and vasodilator cytokines, tilting towards vasoconstriction. Presence of metabolic factors such as obesity, IR and hyperinsulinaemia contributes further to the development of PE, by producing shallower implantation sites, producing further proinflammatory cytokines [36].

The serum ALT is known to rise with the degree of hepatic steatosis among nonpregnant population [37]. Further, the serum ALT level during the third trimester of pregnancy is known to be lower than the nonpregnant women[38]. Our study found that a significant rise of mean ALT level as the degree of hepatic steatosis increase even during pregnancy, still within the normal range, which is a novel finding.

We used USS to identify NAFLD close to delivery, making our study unique in contrast to all the studies mentioned above, where USS was done between 10–14 weeks. Whether pregnancy influences the progression of NAFLD is unknown and recommended for further research. Considering NAFLD is related to diet and bodyweight in nonpregnant women, it could be possible that NAFLD grading may change during the course of pregnancy due to the following; different dietary habits women adopt during pregnancy, emesis gravidarum, natural weight gain and increase in metabolism taking place during pregnancy.

Interest in how altered exposure to excess fuels effect early infant development has intensified in past few years due to recognition that women with obesity or GDM may transmit this phenotype to their offspring. It seems likely that NAFLD, which is the hepatic manifestation of metabolic syndrome, share a developmental origin that begins during pregnancy [39]. In addition, dietary modifications and regular exercises are known to slow down the progress of NAFLD[40]. It is in this light that screening for NAFLD in pregnancy gains significance as lifestyle modifications can be applied to women of child bearing age, with view of improving outcome in subsequent pregnancies. We believe that pregnancy should be used as an opportunity to screen for hepatic steatosis with ultrasonography as most women undergo obstetric ultrasound scans as a part of routine antenatal care.

### Limitations

We used ultrasonography as the tool for identifying NAFLD, as it is the most commonly used and recommended first line imaging modality in clinical practice, where the condition is suspected[40–42]. Main limitation of ultrasonography is the relatively low sensitivity, and specificity. USS has sensitivity of 55–67% and specificity of 77–93% in detecting mild steatosis, while sensitivity of 81–100% and specificity of 98% in recognizing moderate-to-severe steatosis [7]. Though the operator dependency, and less sensitivity especially among morbidly obese subjects remained the main limitations, its availability and proven safety in pregnancy led us to use USS as the diagnostic tool for the study [40,43,44]. We used the standard method described in ‘material and methods’, in identifying the pathology and double checked with the second operator to minimize the inter and intra operator variations. While liver biopsy remains the current ‘gold standard’ clinical assessment tool of NAFLD, it’s use is not justified on asymptomatic women considering the invasive nature and morbidity [45].

We did not have serology of Hepatitis B and C status of the subjects. We excluded hepatitis by history alone. Hepatitis B infection is not common in Sri Lanka and is not considered a major health problem [46]. Studies conducted on different population groups, including blood donors, pregnant mothers, and prison inmates, have shown prevalence of hepatitis B to be less than 2% [47]. A single-center retrospective study involving 696 patients with cirrhosis found only 13 (1.87%) patients had chronic hepatitis B infection [48]. In another study done on 81 patients with cirrhosis who were referred for liver transplantation, none had hepatitis B or C [49]. Even though nationwide data on prevalence of hepatitis C virus (HCV) in Sri Lanka is not available, studies conducted in various specific groups of population have shown a seroprevalence of <1% [46]. Therefore, we assume that not having the serology status would not have made difference to our results. Because of the low prevalence, even the routine antenatal care in Sri Lanka does not have screening for Hepatitis.

Identification of women with past history of diabetes and chronic hypertension was based on patient’s history and record entered at the booking visit by the attending doctor. Sometimes we did not have access to patient’s previous record of the illness concerned. In Sri Lanka, we do not have a computerized medical record and some patients carry their own file.

In calculating the BMI we used the weight at the booking visit and height, rather than the pre-pregnancy values. The median booking period of amenorrhea was between 8–9 weeks in both groups in the study (Table 1). It is well known that the weight gain during first trimester is minimal, thus we do not expect a change in results due to this issue.

Though there is a well-recognized association between obesity, insulin resistance, dyslipidaemia and NAFLD, we did not do glycosylated haemoglobin and lipid profile as a part of the study.

## Conclusion

NAFLD is a common problem during the pregnancy and is associated with the development of gestational hypertension and PE. There was no significant association between NAFLD with GDM, DIP during pregnancy, preterm labour, small for gestational age, caesarean section rate and Apgar of the baby. The mean ALT level increased with the grade of liver steatosis though it remained within the normal range.

## Supporting information

S1 Dataset NAFLD and pregnancy dataset. (SAV)
